# Sheep's Thyroid Gland in the Cure of Sclerodermia

**Published:** 1909-04-03

**Authors:** 


### Dermatology.

### SHEEP'S THYROID GLAND IN THE CURE OF SCLERODERMIA.

Sclerodermia is fortunately a disease that one comes across only now and then, but it is so chronic that the same patient is likely to come under the care of many different medical men at different times. Hence most practitioners have the distressing condition to treat at some time or another. The disease in most cases is slowly progressive, with periods of quiescence. Resolution of the cutaneous and subcutaneous sclerosis is decidedly rare. The following case, therefore, in which a practically complete cure resulted from the administration of sheep's thyroid gland by the mouth cannot but be of interest.

The case was exhibited by M. P. Menetrier before the Société Médicale des Hôpitaux de Paris both in February and in June 1905. In February the sclerodermia was typical and extensive, particularly upon the trunk, neck, and upper limbs, with great limitation in the movements of the latter. Thyroid treatment had already been started at that time, and it was persisted with until June, when the patient was again presented before a meeting of the Society. Fresh sheep's thyroid glands were mashed up in warm soup, a dose of 2 grammes (about half a drachm) being given daily for a week at a time, with interruption of the treatment for alternative periods also of a week. The dosage was always well borne, the only notable symptom having been acceleration of the pulse-rate during the periods when thyroid gland was being taken.

The sclerodermia slowly but progressively lessened. There was no relapse, and not even any cessation in the slow but steady improvement. By June the patient appeared to be cured. There was no longer any tendency to the local erythema after getting warm from muscular effort. The skin where it had formerly been sclerodermic had recovered its softness and suppleness; the arms and neck could be moved about with ease; the patient could walk without fatigue, and there had been an increase of several pounds in the body weight.

Spontaneous resolution of sclerodermia is so rare that the thyroid gland treatment in the above case was in all probability an essential factor in the cure. At any rate, it would seem to be well worth trying in future cases of a similar kind.

